# Non coding extremities of the seven influenza virus type C vRNA segments: effect on transcription and replication by the type C and type A polymerase complexes

**DOI:** 10.1186/1743-422X-5-132

**Published:** 2008-10-30

**Authors:** Bernadette Crescenzo-Chaigne, Cyril Barbezange, Sylvie van der Werf

**Affiliations:** 1 Unité de Génétique Moléculaire des Virus Respiratoires, URA 3015 CNRS, EA 302 Université Paris Diderot, Institut Pasteur, F-75724 Paris, France; 2 UMR 1161 Virologie Afssa Inra Enva, 23 avenue du Général de Gaulle, 94706 Maisons-Alfort cedex, France

## Abstract

**Background:**

The transcription/replication of the influenza viruses implicate the terminal nucleotide sequences of viral RNA, which comprise sequences at the extremities conserved among the genomic segments as well as variable 3' and 5' non-coding (NC) regions. The plasmid-based system for the *in vivo *reconstitution of functional ribonucleoproteins, upon expression of viral-like RNAs together with the nucleoprotein and polymerase proteins has been widely used to analyze transcription/replication of influenza viruses. It was thus shown that the type A polymerase could transcribe and replicate type A, B, or C vRNA templates whereas neither type B nor type C polymerases were able to transcribe and replicate type A templates efficiently. Here we studied the importance of the NC regions from the seven segments of type C influenza virus for efficient transcription/replication by the type A and C polymerases.

**Results:**

The NC sequences of the seven genomic segments of the type C influenza virus C/Johannesburg/1/66 strain were found to be more variable in length than those of the type A and B viruses. The levels of transcription/replication of viral-like vRNAs harboring the NC sequences of the respective type C virus segments flanking the CAT reporter gene were comparable in the presence of either type C or type A polymerase complexes except for the NS and PB2-like vRNAs. For the NS-like vRNA, the transcription/replication level was higher after introduction of a U residue at position 6 in the 5' NC region as for all other segments. For the PB2-like vRNA the CAT expression level was particularly reduced with the type C polymerase. Analysis of mutants of the 5' NC sequence in the PB2-like vRNA, the shortest 5' NC sequence among the seven segments, showed that additional sequences within the PB2 ORF were essential for the efficiency of transcription but not replication by the type C polymerase complex.

**Conclusion:**

In the context of a PB2-like reporter vRNA template, the sequence upstream the polyU stretch plays a role in the transcription/replication process by the type C polymerase complex.

## Background

Type A, B and C Influenza viruses are members of the *Orthomyxoviridae *family. Their genome is segmented and consists of eight segments for type A and B influenza viruses and only seven segments for type C influenza virus that has only one envelope glycoprotein instead of two for the type A and B viruses. Each genomic segment forms a ribonucleoprotein complex (vRNP), composed of a single-stranded RNA molecule of negative polarity (vRNA) associated with molecules of nucleoprotein (NP) and the polymerase complex (P) formed of the PB1, PB2 and PA/P3 proteins.

For each genomic viral RNA, the coding region is flanked by non-coding (NC) sequences at both ends of the segment. These terminal nucleotide sequences are involved in the transcription and replication of viral RNA [1,2] which further require the P and NP proteins. In the nucleus of infected cells, three different RNAs of viral origin are synthesized for each segment. The messenger RNAs (mRNAs) are products of the transcription process. They are capped at the 5' end with a 10 to 13 nucleotides (nt) sequence of nonviral origin derived from newly synthesized host nuclear RNAs through a so-called cap-snatching mechanism. At their 3' end they possess a poly(A) sequence that results from termination of RNA synthesis at a polyU sequence localized 17 to 22 nt upstream of the 5' end of the genomic vRNA template. The full length complementary RNAs of positive polarity (cRNAs) are a product of the replication process and serve as template for the synthesis of genomic vRNAs. Initiation of the synthesis of cRNAs and vRNAs is primer-independent and anti-termination occurs at the polyU sequence during cRNA synthesis (for review [3]).

The NC sequences can be divided into two parts: the conserved and the non conserved sequences [1]. The length of the conserved NC sequences varies between virus types. At the 3' end, the conserved sequence is 12 nt long for type A and B influenza viruses and 11 nt long for type C viruses. At the 5' end, the conserved sequence is 13, 11 and 12 nt long for type A, B and C viruses, respectively [4-6]. The role of the conserved NC sequences has been extensively studied. In cell culture experiments, it was shown that the conserved 3' and 5' NC sequences are sufficient for the expression, the replication and the packaging of the genomic segments [7]. *In vitro *studies suggested that the promoter for transcription is entirely contained within the 3' and 5' NC sequences [8,9], and it was recognized that an interaction between the 3' and 5' NC sequences is required for transcription initiation [10]. Indeed, these conserved NC sequences are partially complementary to each other and have the ability to form partially double-stranded structures involved in the transcription and the replication, in the shape of a panhandle [11-13], or a double hairpin loop or so-called corkscrew structure [14-17].

The role of the polymerase complex in the replication and transcription processes has been mainly studied for type A influenza viruses. The precise domains of interaction between the three polymerase proteins have been defined [18] and their respective role in the transcription and replication processes was analyzed [19]. Type A, B and C influenza viruses share common sequences in the conserved 3' and 5' NC regions of the viral RNA segments. Within each type of influenza viruses and each sub-type of type A influenza viruses, the non conserved NC sequences differ in length and nucleotide composition for the different segments [4,5]. In the case of type A influenza virus, the non conserved NC sequences were shown to modulate the efficiency of transcription and replication [1,2].

We previously showed that type B virus vRNAs could serve as templates for transcription and replication by type A, B and C polymerase complexes [20]. Furthermore, Weber et al. [21] showed that type B but not type A vRNA templates could be used to some extent by the Thogotovirus polymerase complex, another member of the *Orthomyxoviridae *family. However, neither type C vRNA templates, nor type C polymerase complex proteins, were included in their study. We also showed, using only incomplete NC region sequences of the NS segment, that the polymerase complex of type A influenza virus was able to transcribe and replicate vRNA templates from type A, B and C viruses [20]. In contrast, transcription and replication of the reporter vRNA template with type A extremities in the presence of the type C polymerase complex was reduced [20]. Differences in the conserved 3' and 5' NC regions between type A and C vRNAs were shown to contribute to the specificity with which the transcription/replication signals are recognized by the cognate polymerase complexes [14]. To further analyze the type specificity of the interactions between the polymerase complex and the 3' and 5' ends of the vRNA and its consequences on transcription and replication, we extended our study to reporter vRNA templates harboring the complete 3' and 5' NC regions of the seven genomic segments of type C influenza virus.

Firstly, we analyzed the complete 3' and 5' ends of the seven genomic segments of C/Johannesburg/1/66 virus (C/JHB/1/66) which we recently determined [22] and compared the length of the NC sequences of the different genomic segments of the type A, B and C influenza viruses. Then, we generated plasmids that direct the synthesis of reporter vRNA templates harboring the 3' and 5' NC sequences of each of the seven type C influenza virus segments and compared their levels of transcription and replication in the presence of the type C and A influenza virus polymerase complexes using a transient transcription/replication assay [23]. Because this approach based on the CAT reporter gene activity showed major differences for the PB2-like vRNA template, we investigated the sequence requirements for optimal transcription versus replication of the PB2-like template by measuring the levels of mRNA and vRNA by real-time RT-PCR.

## Results and discussion

### Analysis of the complete sequences of the 3' and 5' non coding regions of the genomic RNA segments from influenza virus C/Johannesburg/1/66

In the early 1980s, only partial nucleotide sequences of the 3' and 5' NC regions were determined for the seven genomic segments of C/JHB/1/66 virus by Desselberger et al. [4]. The only complete NC sequences of C/JHB/1/66 in the databases were those of the HEF segment [GenBank: M17868 and AY880247] [24]. In 1984, Clerx-van Haaster and Meier-Ewert [25] also published partial sequences of the 3' NC region for two other type C virus isolates. Overall, very few sequences of the 3' and 5' ends, including complete sequences, are available in the Genbank database. To complete these data and develop a reverse genetic system [22], we determined the complete sequence of the 3' and 5' NC regions of the seven segments of the virus C/JHB/1/66 (Table 1). Since this work was initiated, another group developed a reverse genetic system for type C influenza virus for the C/Ann Arbor/1/50 strain [26]. This required the sequencing of the 3' and 5' NC regions of the seven segments, but until now, these sequences are available in the GenBank database for the NP and NS segments [GenBank: AB126195 and AB283001 respectively] only.

**Table 1:** Sequences of the 3' and 5' NC regions of the genomic segments of C/JHB/1/66 virus.

Segment	3' end non coding sequence^a^
**PB2**	**UCGUCUUCGUC**UC**C**UAACCUU(UAC)
**PB1**	**UCGUCUUCGUC**UC**C**UAA(UAC)
**P3**	**UCGUCUUCGUC**CC**C**UAGGCUU(UAC)
**HEF**	**UCGUCUUCGUC**CC**C**CAAUUAU(UAC)
**NP**	**UCGUCUUCGUC**CU**C**UAAACCAAAAGUUUU(UAC)
**M**	**UCGUCUUCGUC**CC**C**UGAAAAUUUGU(UAC)
**NS**	**UCGUCUUCGUC**CC**C**AUGAAAAAGUUU(UAC)

	5' end non coding sequence^a^
**PB2**	**AGCAGUAGCAAG**AG**G**A*UUU*(*UU*A)
**PB1**	**AGCAGUAGCAAG**AG**G**A*UUUUUU*CAUUUAAUGGAAUAACAAAAAUAUGUGCAAGUAGGAGGAAAGGGUUUAACAGCCCCUCC(UCA)
**P3**	**AGCAGUAGCAAG**GG**G**A*UUUUUU*CUUAUAAUGA(UCA)
**HEF**	**AGCAGUAGCAAG**GG**G**A*UUUUU*GUUUUUUAUAAAACUGUACAAAAUAUUGACCAACACAUUAUCCAUUUUUCAAAAUUGUCUCAA(UCA)
**NP**	**AGCAGUAGCAAG**GA**G**A*UUUUU*GAAUUAUAUAUAGCAAUACAACAGUUGAUCAUAAAAUGUGCGAUGAAUUUAAUCUGACUUUAAUUUUCUCCAGGAAUGUUG(CUA)
**M**	**AGCAGUAGCAAG**GG**G**A*UUUUUU*CAAGGUAA(UUA)
**NS**^b^	**AGCAGGAGCAAG**GG**G***UUUUUU*AACUUUGGAAUAACAACUUAAAACAA(UUA)

^a ^in bold: conserved sequences; in parentheses, start and stop codons; in italic, poly U.

^b ^underlined, variation in the conserved 5' non coding region for the NS segment.

The 3' and 5' NC region sequence of the HEF segment of C/JHB/1/66 were found to be identical to those previously available in the database. Regarding the NC sequences at the 3' ends (Table 1) we observed that, except for the PB1 segment, 1 to 4 nt were missing in the sequences determined by Desselberger et al. [4]. Furthermore, we confirmed that the first 11 nt at the 3' end were conserved for type C influenza virus. Nucleotide 14 was also conserved for the seven segments of the same virus, whereas no nucleotide was conserved at this position between the eight segments of type A influenza virus for which a unique natural variation, U or C, is observed at position 4 [27]. For each type C segment, the optimal context for initiation of translation surrounding the AUG initiation codon was respected at position -3 (i.e. a purine) [28]. This suggests that translation initiation should be efficient for all mRNAs. For type A influenza viruses, a suboptimal Kozak sequence was found at position -3 for the PB1 and NA mRNAs and was shown to alter translation efficiency when using a reporter gene but not in the context of infectious virus [29].

The results obtained for the 5' ends showed that the data of Desselberger et al. [4] only covered part of the NC sequences corresponding to the first 11 to 23 nt from the extremities. Indeed, for the PB2, P3 and M segments, the 5' NC sequences were three to seven nt longer, whereas for the PB1, HEF, NP and NS segments, the actual 5' NC sequences were 25 to 86 nt longer than those published by Desselberger et al. [4]. We confirmed that the first 12 nt of the 5' end were conserved among the seven segments and, contrary to type A influenza virus, nucleotide 15 was also conserved. However, for the NS segment 5'end, we noticed one difference at nt 6, for which the U residue was replaced by a G. This was already described for the 5' end of the NS segment of the C/California/78 and C/Ann Arbor/1/50 viruses [GenBank: M10087 and AB283001 respectively] and might then represent a specific feature of the type C influenza virus NS segment. Noticeably, the length of the 5' NC sequences of type C virus was very variable among the different segments, ranging from 19 nt for the PB2 segment to 102 nt for the NP segment (excluding the start and stop codons). The virus utilized the 3 different stop codons: (UAA) for PB2, M and NS; (UGA) for PB1, P3 and HEF and (UAG) for NP. It is also noteworthy that, for the segment with the shortest 5' NC sequence, i.e. the PB2 segment, the polyU overlapped the stop codon. The polyU stretch for all segments was found to be 5 or 6 U residues long, which was described as an optimal length for the polyadenylation of mRNAs for type A influenza virus [30]. We found a polyU of 5 residues for the PB2, HEF and NP segments, and of 6 residues for the other segments. The length of the polyU stretch was identical for counterpart segments of type A influenza virus A/PR8/34. For the HA and NA segments of this virus, the polyU stretch was 5 and 6 nt long, respectively (data not shown).

Finally, we noted that nucleotides 1 to 4 and 9 to 12 of the conserved 5' NC sequence of Thogotovirus [31], a distantly related member of the *Orthomyxoviridae*, were identical to those found in the conserved 5' NC region of type C influenza virus but differed from that of type A viruses.

### Comparison of the lengths of the 3' and 5' non coding sequences of the genomic segments of type A, B and C influenza viruses

To compare the lengths of the 3' and 5' NC sequences of the different genomic segments of type A, B and C influenza viruses, we used our own sequencing results for C/JHB/1/66 virus [22] and several sequences retrieved from the GenBank database for viruses isolated between 1933 and 2005. We used only sequences for which the complete 3' and 5' NC regions were available. Consequently, the number of sequences used for comparison was variable for each virus type (or segment), with obviously much more information available for the numerous sub-types of type A influenza virus. One should note that, prior to this report, no sequences of the 3' and 5' NC regions were available in the Gene Bank database for all segments from one given strain of type C influenza virus, which is still the case for type B influenza virus. A list of all the sequences used with their access numbers is available from the authors upon request.

As shown in Table 2, important variations in the length of the NC sequences were observed between the eight genomic segments of type A and B influenza viruses and the seven segments of type C influenza viruses. The length of the 3' NC sequences ranged from 19 to 45 nt, 21 to 58 nt, and 17 to 29 nt for type A, B and C influenza viruses, respectively. The 3' NC sequences of type C virus appeared more homogeneous in their length and generally shorter than those of type A and B viruses, the length range of 3' NC sequences of type B virus being the widest.

**Table 2:** Length of type A, B and C influenza virus 3' and 5' NC sequences

**Type A**	**PB2**	**PB1**	**PA**	**HA**	**NP**	**NA**	**M**	**NS**
3'NC	27	24	24	*	45	**	25	26
5'NC	34	43	58	*	23	**	20	26

*	**H1**	**H2**	**H3**	**H5**		**	**N1**	**N2**

3'NC	32	43	29	28		3'NC	20^a^	19
5'NC	45	41	32–35	44		5'NC	28	38

								

**Type B**	**PB2**	**PB1**	**PA**	**HA**	**NP**	**NA**	**M**	**NS**
3'NC	23	21	29	33	58	53	24	42
5'NC	60	89	98	94	101	103	91	30

								

**Type C**	**PB2**	**PB1**	**P3**	**HEF**	**NP**	**M**	**NS**	
3'NC	21	17	21	21	29	25	26	
5'NC	19	81	32	84	80–102^b^	30	47	

Lengths are in nt, the start and stop codons were excluded.

^a^: except for A/WSN/33 which has 19 nt

^b^: 80 nt for C/Ann Arbor/1/50, 82 nt for C/California/78, 102 nt for C/JHB/1/66

The longest 3' NC sequence of all segments was found for the NP segment for the 3 types of influenza viruses. It remains to be determined whether the length of the 3' NC region might have an influence on the transcription and/or replication of that particular segment and consequently on the level of expression of the NP protein, which might be in relation with the important functions of this protein in the regulation of transcription and replication and in the encapsidation of the viral RNAs [32,33].

The length of the 5' NC sequences ranged from 20 to 58 nt, 30 to 103 nt, and 19 to 102 nt for type A, B and C influenza viruses, respectively, the length of the 5' NC regions of type B and C viruses being more heterogeneous than those of type A influenza virus (Table 2). Among the type A viruses, it is interesting to note that the 5' NC sequences of the HA segment of human H3 viruses differed between strains isolated before and after 1977. Viruses isolated from 1978 onward were characterized by a 3 nt deletion within the non conserved 5' NC sequences, and the total length of the 5' NC region was reduced from 35 to 32 nt. This reduction of the length of the 5' NC sequence was never observed for H3 isolates of avian or equine origin. Interestingly, in swine, co-circulation of H3 isolates with either 35 or 32 nt long 5' NC sequences is observed in agreement with the human origin of some swine viruses (data not shown). Another point worth noticing regarding type A influenza concerned the 5' NC sequence of the PB1 segment. Two adjacent stop codons were found in most strains we analyzed. However, for a few strains of different subtypes of avian origin isolated from humans or birds, the first stop codon was mutated into a coding codon (data not shown). Whether the resulting variation at the C-terminus of the PB1 protein might have functional consequences remains to be determined.

Only three complete sequences of full-length NP segments are available for the type C viruses, i.e. C/California/78 [GenBank: M17700], C/Ann Arbor/1/50 [GenBank: AB126195] and C/JHB/1/66 [GenBank: AF170573] and were found to be 1809, 1807 and 1802 nt long, respectively. The 7, 5 and 2 nt discrepancies are related to differences in the 5' NC region. Indeed the C/JHB/1/66 like the C/Ann Arbor/1/50 NP segment harbors the same 2 nt deletion in the 5' NC region as compared to C/California/78. Moreover, because of an additional 5 nt deletion for the C/JHB/1/66 strain, the NP ORF was shifted, resulting in the use of a different stop codon. Thus, the NP protein was 9 amino acids shorter for C/JHB/1/66 than for C/California/78 and C/Ann Arbor/1/50 and had a C-terminal Lys residue replacing a Ser, 10 residues from the C-terminus of C/California/78 and C/Ann Arbor/1/50 (data not shown). It is necessary to sequence more strains of type C virus to evaluate the variability in the length of the NP. Since the C-terminus of type A influenza virus NP is implicated in the homo-dimerisation of NP [34] and in the interaction with PB2 [35,36], it would be of interest to study the influence of such variability on the NP-PB2 interaction in a type C context.

When looking at the data of the type A viruses, we also noted that for a given segment (for HA and NA segments, within a specific subtype) the length of both NC sequences were conserved among human, avian, swine, equine and any other species isolates (data not shown). This suggests that the length of the NC regions is not a limiting factor for the occurrence of reassortment events between viruses of different host origin. It is not known however whether specific nucleotides within the NC regions could be involved in species-specificity. It has been demonstrated that sequences required for efficient packaging of influenza virus genomic segments involved both non coding and coding regions [37-43]. However, the precise role of the NC regions and to what extent the length of these NC regions might be important for the packaging process remains to be determined. Because the 5' NC regions of type C and type B are much longer than for type A viruses, possible length involvement could be studied more easily for these virus types.

### Activity of the type C and A polymerase complexes on viral-like RNA templates derived from the seven genomic segments of type C influenza virus

Here we further studied the efficiency with which virus-like vRNAs harboring the full-length NC regions of each of the seven segments of C/JHB/1/66 virus could be used as templates for the transcription and replication by both type C and A virus polymerase complexes. Analysis was performed using a transient transcription/replication assay based on CAT expression, as described in Methods.

**Figure 1:**
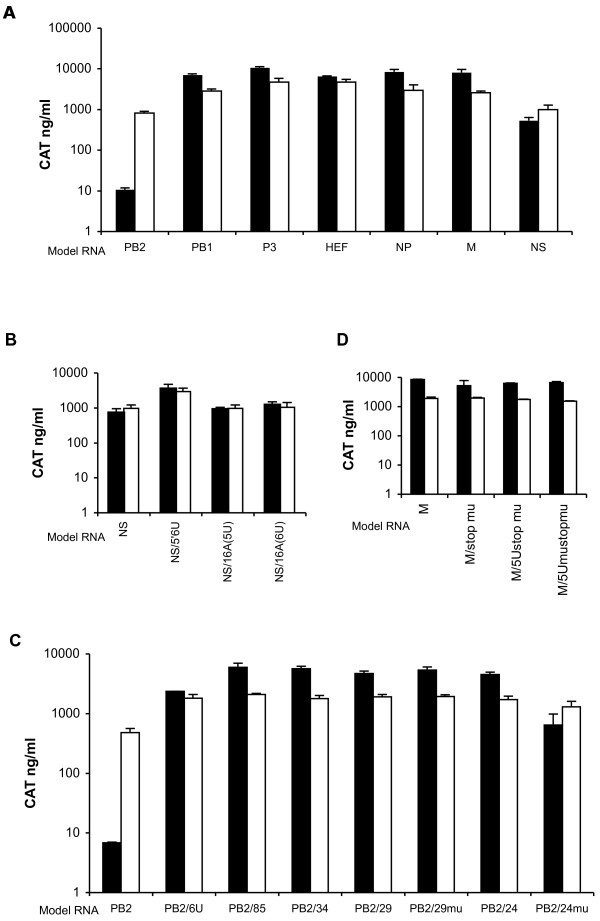
**Transcription/replication of (-) sense model RNA templates derived from the type C genomic segments**. 293T cells were transfected in duplicate with the four pHMG plasmids encoding the polymerase complex of influenza virus type C (closed bars) or type A (open bars), together with 100 ng of plasmids expressing CAT reporter vRNA-like templates derived from the influenza virus type C genomic segments as indicated. At 24 h post-transfection, cell extracts were prepared and the levels of CAT were determined as described in Methods. The results are expressed as the mean +/- SD of duplicate samples from one experiment representative of two independent experiments for A, C and D and as the mean +/- SD from two experiments for B. The names of the pC/PRCAT/plasmids were shortened to the name of the virus segment or of the mutant. (A) vRNA-like templates with wild-type NC regions. (B-C-D) respectively, NS-, PB2-, M- like vRNA templates with mutations in the 5'NC sequence.

As shown in Fig. 1A, similar levels of CAT expression were obtained for PB1, P3, HEF, NP and M vRNA templates with both type C and A polymerase complexes. Although no significant difference was noted, the CAT levels obtained with the type C polymerase complex were slightly higher than those with the type A complex, suggesting a better efficiency in a homologous context. Maeda et al. [29] did not find any difference in the reporter gene expression levels in a similar experiment based on virus-like vRNAs and polymerase complex of type A virus. The only differences they observed for PB1 and NA segments were indeed attributed to variations in translation efficiency resulting from a suboptimal Kozak sequence. As mentioned earlier the differences in the length of type A virus NC regions among the eight segments are smaller than those observed among the seven type C segments (Table 2). However, our results using type C vRNA templates confirm that the length of the 5' NC region up to 102 nt (NP segment) did not influence the levels of transcription/replication by the type A polymerase. With the type C polymerase no significant variations were observed for the PB1, P3, HEF and NP vRNA templates, again indicating that variations in the length of the 5'NC sequence from 30 to 102 nt did not influence the levels of transcription/replication. In contrast, in the context of the Uukuniemi virus, another negative-stranded segmented RNA virus, it was shown that the longer the 5' NC region, the higher the level of the reporter gene expression, demonstrating the influence of the length on transcription/replication efficiency [44].

The NS vRNA template was used by both type C and A polymerase complexes with the same efficiency (Fig. 1A). However, with the type C polymerase complex, the level of CAT expression for the NS template was found to be 10 fold lower than for PB1, P3, HEF, NP and M vRNA templates. As mentioned earlier, the C/JHB/1/66 virus NS segment has a G residue at nucleotide 6 in the conserved 5' NC region instead of the usual U found for all the other segments (Table 1). When a substitution was introduced at nt 6 in the 5' NC region of the NS template to replace the G residue by a U (NS/5'6U, Table 3), the transcription/replication levels were found to be 5-fold higher than for the NS wt vRNA template (Fig. 1B), showing that the conserved 5'NC region directly influenced the transcription/replication.

**Table 3:** Sequences of the 5' NC region of the mutated NS-, PB2- and M-like vRNA templates

Segments and mutants	sequences
NS	AGCAGGAGCAAGGGGUUUUUUAACUUUGGAAUAACAACUUAAAACAA**UUA**
NS/5'6U	AGCAGUAGCAAGGGGUUUUUUAACUUUGGAAUAACAACUUAAAACAA**UUA**
NS/16A(5U)	AGCAGGAGCAAGGGGAUUUUU AACUUUGGAAUAACAACUUAAAACAA**UUA**
NS/16A(6U)	AGCAGGAGCAAGGGGAUUUUUUAACUUUGGAAUAACAACUUAAAACAA**UUA**

PB2	AGCAGUAGCAAGAGGAUUU**UUA**
PB2/6U	AGCAGUAGCAAGAGGAUUUU**UUA**
PB2/85	AGCAGUAGCAAGAGGAUUU**UUA**GUUAGACAUCUUUAUCUUUUUCACAUUCUUAUUUACAUCGCUUGAUGCA GCCCUUUGUGAGGC**UUA**
PB2/34	AGCAGUAGCAAGAGGAUUU**UUA**GUUAGACAUCUU**UUA**
PB2/29	AGCAGUAGCAAGAGGAUUU**UUA**GUUAGAC**UUA**
PB2/29 mu	AGCAGUAGCAAGAGGAUUU**UUA**GCAAGAC**CUA**
PB2/24	AGCAGUAGCAAGAGGAUUU**UUA**GU**UUA**
PB2/24 mu	AGCAGUAGCAAGAGGAUUU**UUA**CG**CUA**

M	AGCAGUAGCAAGGGGAUUUUUUCAAGGUAA**UUA**
M/stop mu	AGCAGUAGCAAGGGGAUUUUUUCAAGGUAA**CUA**
M/5Ustop mu	AGCAGUAGCAAGGGGAUUUUU CAAGGUAA**CUA**
M/5Umustop mu	AGCAGUAGCAAGGGGAUUUUU CAAGGCAA**CUA**

In bold, stop codons. Underlined, introduced mutations or deletions.

A heterogeneity at nucleotide 6 among the conserved 5' NC regions of the different segments was also observed for type B influenza virus [6]. Only few studies on the transcription and replication levels of type B virus segments have been published [45-47], but none on the role of this particular position in the conserved 5' NC region. For type A influenza virus, it has been shown that nucleotides, in the conserved 5' NC region, but not nucleotide at position 6, are critical for transcription/replication of the vRNA [15,16].

In a similar transient transcription/replication assay using the CAT reporter gene, Li and Palese [30] showed that for type A influenza virus the length of the NC sequence between the polyU stretch and the 5' extremity influenced the level of CAT expression. Indeed they found that the levels of CAT expression were different between NS and NA vRNA templates. This could be correlated with the difference in distance of the polyU stretch from the 5' extremity, which was 16 and 15 nt long for NS and NA segments, respectively. When elongating the NA sequence by one nt to reach a distance similar to that of the NS segment, CAT expression was increased two-fold. On the contrary, shortening the NS sequence by one nt dramatically reduced CAT levels. On the other hand, inserting two nt in the NA sequence or one nt in the NS sequence completely abolished CAT expression. The authors thus concluded that the optimal distance between the polyU stretch and the 5' extremity was 16 nucleotides [30]. Interestingly, this distance of 16 nt was also found for all the segments of type C influenza virus C/JHB/1/66 except for the NS segment, which was characterized by only 15 nucleotides (Table 1). However, this length proved to have no influence on the level of transcription/replication, since a 16 nt long conserved 5' NC end, generated in NS16A(6U), did not increase the CAT level in transient expression assays (Fig. 1B), even when the total length of the 5' NC end was conserved (Table 3: NS16A(5U)).

More striking observations were made for the PB2-like vRNA template. Whereas with the type A virus polymerase complex the level of CAT expression was similar to that observed with the six other segments, with the type C virus polymerase, it was reduced nearly 1000-fold, when compared to the level obtained with the P3-like vRNA template (Fig. 1A). As noted earlier, the 5' NC sequence of the type C influenza virus PB2 segment is the shortest among all segments of the three types of influenza viruses (Table 2). Furthermore, the stop codon of the PB2 ORF overlaps the polyU stretch (Table 1). In order to determine whether the absence of a non conserved NC region between the stop codon and the polyU stretch might have an effect on the level of transcription/replication of the type C virus PB2-like vRNA template, we constructed several mutant templates (Table 3). As shown in Fig. 1C, in the presence of the type A polymerase complex, similar levels of CAT expression were observed for wild-type and mutant PB2-like templates. This confirmed that the type A polymerase complex is not affected by variations in the NC regions, in agreement with its ability to act as a universal polymerase complex for all types of influenza viruses [20].

The PB2/6U RNA template contained an additional U in the polyU stretch. Indeed for type C influenza virus segments, the polyU stretch is 6 nt long except for the PB2, HEF and NP segments. For type A influenza virus, a 5 nt long polyU was shown to be efficient as already mentioned [30]. In the presence of the type C polymerase complex, the CAT expression level was increased about 100-fold when an additional U was introduced into the polyU stretch (PB2/6U; Fig. 1C). Several mutant templates PB2/85, 34, 29 and 24 (Table 3) were then designed to study the influence of an extension of 64, 13, 8, or 3 nt respectively between the polyU stretch and the stop codon of the CAT open reading frame to mimic a putative non conserved 5'NC region. We chose to use sequences corresponding to the end of the PB2 coding sequence. For all these mutants, the CAT expression level was more than 500-fold higher than that for the wt PB2-like vRNA template (Fig. 1C), nearly reaching the levels obtained for the M-like vRNA template (data not shown). For the PB2 segment, 3 additional nt upstream the polyU thus appeared to be necessary and sufficient to restore levels of transcription/replication comparable to those of the other segments. It was striking to note that the 5' coding sequence of the PB2 segment of type C influenza virus is rich in U residues, which prompted us to generate mutants PB2/29 mu and 24 mu to test whether the presence of U residues upstream of the polyU was important for restoration of efficient transcription/replication (Table 3). As shown in Fig. 1C, substitution of the U residues had no influence in the case of PB2/29 mu. Similarly, in the context of a M-like vRNA template, reduction of the number of U residues in the non conserved 5' NC region had no influence on the transcription/replication levels (Table 3 and Fig. 1D). However, the CAT expression level observed with the PB2/24 mu template was 10-fold lower than with the PB2/24 template (Fig. 1C). Thus, it appeared that optimal transcription/replication of the vRNA is observed when a minimum of 8 nt of the non conserved 5' NC region is maintained between the polyU stretch and the stop codon (PB2/29 mu and M/5U mustopmu). However, in the case of the PB2 segment, further shortening of the non conserved 5'NC sequence that resulted in reduced transcription/replication efficiency could be compensated by the presence of several U residues upstream of the polyU stretch (PB2/24).

### Analysis of the vRNA and mRNA levels produced from type C PB2-like vRNA templates

To try to understand which of the transcription or the replication steps was more affected by variations in the sequence of the 5' NC region of the type C virus PB2 segment, we analyzed the respective levels of mRNA and vRNA produced from the PB2 and mutant templates by real time RT-PCR targeting the CAT reporter gene, as described in Methods.

The Ct-values shown in Table 4 are the mean of 6 experiments. No Ct value could be assigned when no reverse transcriptase was used during the reverse transcription step, indicating that RNA samples were not contaminated by plasmid DNA (data not shown). When the vRNA template plasmids were co-transfected with the empty pHMG plasmid, i.e. in the absence of polymerase complex plasmids, the Ct values were all above the last value of confidence (Ct = 33) obtained for the standard dilution series and the samples were considered as negative.

**Table 4:** Analysis of the CAT vRNA and mRNA levels by real-time RT-PCR

Polymerase complex^a^	vRNA template^b^	Ct vRNA^c^	Ct mRNA^c^
none	PB2	34.2 ± 0.7	38.4 ± 1.5
	PB2/6U	34.6 ± 1.1	39.0 ± 1.4
	PB2/24	33.6 ± 0.6	39.2 ± 0.6
	PB2/24 mu	32.9 ± 0.7	37.8 ± 1.1
	M	33.5 ± 1.0	37.9 ± 0.8

Type C	PB2	20.5 ± 0.6	**23.0 ± 1.1**
	PB2/6U	18.6 ± 1.4	18.3 ± 0.6
	PB2/24	20.0 ± 0.5	19.8 ± 1.3
	PB2/24 mu	19.1 ± 0.6	20.4 ± 0.8
	M	19.3 ± 1.2	19.5 ± 1.5

Type A	M	19.6 ± 1.1	19.5 ± 1.4

^a^: 293T cells were transfected in duplicate either with plasmid pHMG alone (no polymerase complex) or with the four pHMG plasmids encoding the polymerase complex of type C or type A influenza virus.

^b^: vRNA templates were derived from the M or PB2 (with wt or mutated 5' non coding sequences) segments of the influenza virus type C.

^c^: Values are means ± SD of six independent cDNA syntheses from two independent transfections (three cDNAs per transfection).

According to the CAT expression results (Fig. 1A), we used as a control the M-like vRNA template, characterized by the shortest 5' NC sequence after the PB2-like template. As expected, the levels of transcription and replication were similar with both type C and A polymerase complexes in the case of the M-like template (Table 4). In the presence of the type A polymerase complex no significant differences were observed in the levels of both mRNA and vRNA between the various virus-like vRNA templates as observed with the M-like template (data not shown). This is in agreement with the CAT expression results (Fig. 1A) and confirmed our previous results [14,20] and the fact that the length of NC regions do not influence the activity of the type A polymerase complex on type C virus segments.

In the presence of the type C polymerase complex, the vRNA levels were similar for the PB2-like template and the derived mutants (Table 4). In contrast, a significant reduction in the mRNA levels (p-values < 0.002) was observed for the wild type PB2-like template when compared to each of the three mutants tested (Table 4). No significant difference was observed in the mRNA levels between any of the three mutant templates. Thus the higher levels of CAT expression observed for the mutants were most likely due to improvement of the efficiency of the mRNA synthesis, most likely at the termination/polyadenylation step, whereas there was little or no impact on the efficiency of replication.

## Conclusion

Here we presented the analysis of the sequences of the 3' and 5' NC regions of the C/JHB/1/66 strain. We were intrigued by the range in length of these regions among the seven genomic segments, particularly at the 5' ends, and decided to study their influence on the transcription and replication of each segment. We based our study on the commonly used transient transcription/replication assay of viral-like reporter RNA templates. The transcription/replication efficiency by the type A polymerase complex was not influenced by the length of the non conserved NC sequences at the 3' or 5' ends of the RNA template. For the type C polymerase complex, the transcription/replication efficiency was not significantly influenced by the length of the non conserved NC sequences but a minimum length at the 5' end seemed to be required as shown when analyzing the PB2-like template. Interestingly, for the PB2-like template which lacks non conserved 5' NC sequences the nature of the sequence upstream of the polyU stretch and in particular the presence of U residues was found to be important. Moreover, such sequence requirements were essential for the transcription process whereas no significant effect could be detected for the replication process. In addition, for the NS segment a G residue was found at nt 6 in the conserved 5'NC region instead of a U found for all other segments, which accounted for a reduced transcription/replication efficiency of the NS viral-like RNA template. To what extent the presence of a G rather than a U residue at nt 6 in the 5'NC sequence of the NS segment is important for virus multiplication remains to be determined. The reverse genetics system for type C influenza virus [22] should prove particularly useful to determine the importance in the context of viral multiplication of the sequence requirements identified in this study.

## Methods

### Plasmids for the expression of viral proteins

Plasmids A-pHMG-PB1, -PB2, -PA and -NP, which express the PB1, PB2, PA and NP proteins, respectively, of influenza virus A/Puerto Rico/8/34 (PR8) under the control of the hydroxymethylglutaryl coenzyme A reductase (HMG) promoter were kindly provided by J. Pavlovic (Institut für Medizinische Virologie, Zurich, Switzerland). The construction of the analogous C-pHMG-derived plasmids encoding the -PB1, -PB2, -P3 and -NP proteins of the virus C/JHB/1/66 have been described previously [20].

### Plasmids for the expression of virus-like RNAs

The pPR plasmid vector, in which *Bbs*I restriction sites are flanked at the 5' end by the human PolI promoter and at the 3' end by hepatitis delta ribozyme sequences was described previously [20]. Seven constructs comprising the CAT gene sequence in an antisense orientation flanked by the 5' NC (including the stop codon) and 3' extremities of the seven genomic segments inserted at the *Bbs*I site of the pPR plasmid vector were produced. To generate the CAT sequences flanked with NC sequences corresponding to each of the vRNA segments, the following strategy was used: primers to amplify the CAT gene from pC/PRCAT [20] were designed to include the respective 3' and 5' NC sequences of each of the segments and a *Bbs*I-compatible restriction enzyme site. In the case of the PB1, HEF and NP segments, the primers including the 5' NC sequences were produced by RT-PCR using vRNA as a template and purified before use to amplify the CAT gene. Insertion of the amplification products, at the *Bbs*I site of plasmid pPR resulted in plasmids pC/PRCAT/X where X corresponds to each of the segments, respectively. Plasmids analogous to pC/PRCAT/PB2 and harbouring mutations in the PB2 5' NC sequence were generated using the same strategy. The pC/PRCAT/PB2/6U varies from pC/PRCAT/PB2 by one additional T in the sequence corresponding to the polyU stretch. In plasmids pC/PRCAT/PB2/85, pC/PRCAT/PB2/34, pC/PRCAT/PB2/29, and pC/PRCAT/PB2/24 the 5' NC sequence is extended by 66, 15, 10 nt and 5 nt respectively upstream the regular stop codon including an additional stop codon (see Table 3). Mutations in the pC/PRCAT/PB2/29, pC/PRCAT/PB2/24, pC/PRCAT/M and pC/PRCAT/NS plasmids (Table 3) were introduced using the Quikchange II site-Directed mutagenesis kit (Stratagene) according to the manufacturer's instructions. The sequence of all primers used can be obtained from the authors upon request. Clones with proper inserts or obtained by mutagenesis were checked by sequencing, using a Big Dye terminator sequencing kit and an automated sequencer (Perkin-Elmer). The names of the pC/PRCAT/plasmids were shortened to the name of the virus segment or of the mutant.

### Transfections and CAT assays

293T cells were grown in Dulbecco's modified Eagle's medium containing 10% fetal calf serum. Cells were maintained at 37°C with 5% CO_2_. For each virus-like vRNA template, subconfluent monolayers (9 × 10^5 ^cells in 35-mm dishes) were transfected using 10 μL of FUGENE 6 (Roche) with 100 ng of the corresponding pC/PRCAT plasmid and the four pHMG-derived plasmids coding for the NP protein (2 μg) and the polymerase complex proteins (1 μg) of type C or A influenza virus. Plasmid pHMG alone was used as negative control. Cells were incubated at 37°C for 24 h post-transfection. Using the CAT ELISA Kit (Roche), CAT levels were tested in cell extracts prepared in 500 μL of the lysis buffer provided by the kit. This procedure allows detection of 0.05 ng/mL CAT.

### RNA extraction and Real time PCR

293T cells were transfected as previously, but with only 0.1 ng of the different pC/PRCAT plasmids. Twenty-four hours post-transfection, cultures were washed twice with PBS and total RNA was extracted using the Nucleospin RNA II kit (Macherey-Nagel). The totality of the extracted RNAs (60 μL) was digested with two units of Turbo DNAse (Ambion) at 37°C for 1 hour to eliminate traces of transfected plasmid DNA according to the manufacturer's instructions. The vRNA and mRNA of the CAT gene were reverse transcribed using AMV reverse transcriptase (Promega) with primer 6 s and oligo dT, respectively. The cDNA template (5 μL) was next amplified in MicroAmp Optical 96-well reaction plates in 50 μL of 1× "Master Mix Reaction Buffer" (Eurogentec) in the presence of the CAT specific primers 6 s and 5 as (300 nM each) and of a CAT specific fluorogenic probe (100 nM) labeled with 6-carboxyfluorescein (FAM) and 6-carboxytetramethylrhodamine (TAMRA) at the 5' and 3' ends, respectively. The set of primer and probe sequences for detection of the CAT RNA were as follows: sense CAT primer (6 s) 5'-GCTGGATATTACGGCCTTTTTAAA-3'; antisense CAT primer (5 as) 5'-ACCGTCTTTCATTGCCATACG-3'; and CAT probe 5'(FAM)-TATTCACATTCTTGCCCGCCTGATGAA-(TAMRA)3'. Primers and probe sequences were determined with the PrimerExpress software (version 1.5; Applied Biosystem). After Uracil-DNA Glycosylase (UNG) treatment at 50°C for 2 min and UNG inactivation at 95°C for 5 min, the cycling conditions were 15 s at 95°C, 1 min at 60°C for 40 cycles. Quantification was performed with the ABI PRISM 7700 sequence detection system. A serial dilution of pC/PRCAT plasmid DNA [20], ranging from 10^7 ^to 10^1 ^DNA copies per reaction, was included on each 96-well plate. Results were expressed as Ct values since the DNA standard curve was not reverse transcribed. The Student's *t *test was used to compare the results of the real-time RT-PCR, using 6 replicates for each point.

## Competing interests

The authors declare that they have no competing interests.

## Authors' contributions

BCC performed the experiments, analyzed the results and drafted the manuscript. CB took part in the analysis of the results and in the writing of the manuscript. SW supervised all phases of the project: conception and design of the experiments, analysis of the results and writing of the manuscript. All authors read and approved the final manuscript.
